# Investigating the role of Hedgehog/GLI1 signaling in glioblastoma cell response to temozolomide

**DOI:** 10.18632/oncotarget.25467

**Published:** 2018-06-05

**Authors:** Jilian R. Melamed, Joshua T. Morgan, Stephen A. Ioele, Jason P. Gleghorn, Jennifer Sims-Mourtada, Emily S. Day

**Affiliations:** ^1^ Biomedical Engineering, University of Delaware, Newark, DE, USA; ^2^ Bioengineering, University of California, Riverside, CA, USA; ^3^ Biological Sciences, University of Delaware, Newark, DE, USA; ^4^ Helen F. Graham Cancer Center and Research Institute, Newark, DE, USA; ^5^ Materials Science and Engineering, University of Delaware, Newark, DE, USA

**Keywords:** glioblastoma, hedgehog, GLI1, temozolomide, chemoresistance

## Abstract

Resistance to chemotherapy substantially hinders successful glioblastoma (GBM) treatment, contributing to an almost 100% mortality rate. Resistance to the frontline chemotherapy, temozolomide (TMZ), arises from numerous signaling pathways that are deregulated in GBM, including Hedgehog (Hh) signaling. Here, we investigate suppression of Hh signaling as an adjuvant to TMZ using U87-MG and T98G cell lines as *in vitro* models of GBM. We found that silencing GLI1 with siRNA reduces cell metabolic activity by up to 30% in combination with TMZ and reduces multidrug efflux activity by 2.5-fold. Additionally, pharmacological GLI inhibition modulates nuclear p53 levels and decreases MGMT expression in combination with TMZ. While we surprisingly found that silencing GLI1 does not induce apoptosis in the absence of TMZ co-treatment, we discovered silencing GLI1 without TMZ co-treatment induces senescence as evidenced by a significant 2.3-fold increase in senescence associated β-galactosidase staining, and this occurs in a loss of PTEN-dependent manner. Finally, we show that GLI inhibition increases apoptosis in glioma stem-like cells by up to 6.8-fold in combination with TMZ, and this reduces the size and number of neurospheres grown from glioma stem-like cells. In aggregate, our data warrant the continued investigation of Hh pathway inhibitors as adjuvants to TMZ chemotherapy and highlight the importance of identifying signaling pathways that determine whether co-treatment will be successful.

## INTRODUCTION

Glioblastoma (GBM) is the deadliest form of brain cancer and represents the most common central nervous system tumor in adults, accounting for 45.6% of all malignant primary brain tumors [1]. Standard treatment for newly diagnosed primary GBM includes surgical resection, radiation, and chemotherapy [2, 3]. Temozolomide (TMZ) is the current frontline chemotherapeutic for GBM and acts as an alkylating agent to induce DNA damage and trigger cell death. While the addition of TMZ chemotherapy to the standard radiotherapy has increased median patient survival time from 12.1 months to 14.6 months, tumor recurrence remains virtually inevitable, and nearly 100% of patients ultimately succumb to disease [4]. GBM recurrence is driven in large part by intrinsic or acquired chemoresistance [5], and tumors that have recurred often demonstrate enhanced resistance to chemotherapy and are more invasive than the primary lesion [2].

GBM drug resistance is mediated both by signaling mechanisms throughout the tumor and by an aggressive subpopulation of tumor cells known as glioma stem-like cells, or GSCs. In both GSCs and differentiated GBM cells, deregulated DNA damage repair mechanisms oppose TMZ-mediated cytotoxicity by repairing DNA damage caused by TMZ to prevent apoptosis [6, 7]. In particular, upregulated MGMT, an enzyme that removes alkyl groups transferred to DNA by TMZ, is a known prognostic indicator of poor response to TMZ [8]. Additionally, mutations to the p53/Mdm2/PTEN tumor suppressor axis promote cell cycle progression to suppress TMZ-induced apoptosis [9, 10]. Further, upregulation of EGFR signaling through wild-type EGFR and the commonly expressed EGFRvIII mutant stimulate survival signaling through the Ras/Raf/MAPK and PI3k/Akt/mTOR pathways to promote TMZ resistance [11, 12]. In parallel, TMZ resistance is provided by a subpopulation of multipotent, slow-cycling GSCs that evade chemotherapeutic apoptosis and repopulate the tumor with resistant cells following treatment [13–15]. While much remains unknown regarding their biology, these cells may be identified by various markers (CD133, CD44, nestin, ALDH1, Oct4, Sox2), and differential expression of these markers suggest a stemness hierarchy that may correlate with aggressiveness [16]. Elimination of GSCs remains a barrier to complete tumor eradication.

A growing body of evidence attributes tumor resistance phenotypes and the maintenance of GSCs to deregulated developmental pathways, such as Notch, Wnt, and Hedgehog (Hh) signaling [17–21]. In particular, research has demonstrated that while several developmental pathways contribute to GBM progression, Hh signaling is indispensable for GSC proliferation and tumorigenesis [19] and may be an impactful target for new GBM treatment strategies. Hh signaling is a highly conserved developmental pathway that is normally minimally active in differentiated adult tissue, but also plays an impactful role in the progression and maintenance of several cancers, including GBM. Hh signaling is activated when Hh ligands, most notably sonic hedgehog (Shh), bind the extracellular domain of the Patched (Ptc) transmembrane receptor. Ptc then relieves its suppression of Smoothened (Smo), a second transmembrane protein, which in turn initiates an intracellular signaling cascade that translocates members of the GLI family of zinc finger transcription factors to the nucleus to regulate the expression of target genes (Supplementary Figure 1). Within this family of transcription factors, upregulated GLI1 expression and transcriptional activity is associated with poor patient prognosis in several cancers [22]. Further, GLI1 is known to regulate proliferation and cell cycle progression [23, 24], cell survival [24, 25], migration and invasion [26], cancer cell stemness and self-renewal [23], and response to chemotherapy [18, 23, 27].

In this study, we investigated the role of Hh/GLI1 signaling in GBM resistance to TMZ. Specifically, we evaluated the molecular and phenotypic consequences of GLI1 inhibition as an adjuvant to TMZ treatment to assess the therapeutic potential of this co-treatment strategy. In this work, we used U87-MG and T98G cells as established *in vitro* models of GBM. These models were chosen because they both exhibit active Hh signaling as indicated by GLI1 expression and nuclear localization, but they differ in the expression of known molecular contributors to TMZ resistance. For example, U87-MG cells express wild-type p53, while T98G cells express a mutant p53 variant [9]. Although the role of p53 variants in GBM are not fully understood, evidence suggests that wild-type p53 generally retains tumor suppressive functions, while mutant p53 may promote tumor progression [9, 28, 29]. Additionally, T98G cells, but not U87-MG cells, express high levels of MGMT, which is a primary mechanism by which GBM cells resist alkylating chemotherapies [9, 30, 31]. Because MGMT contains a GLI1 binding domain and consequently may be regulated by Hh signaling [32], MGMT expression may influence GBM cell response to co-treatment with Hh/GLI1 inhibitors and TMZ. Thus, we aimed to capture these key phenotypic differences characteristic of GBM resistance mechanisms with our choice of established cell models.

Here, we show that silencing GLI1 prior to treating cells with TMZ increases the cytotoxicity of TMZ against GBM cells. We provide additional evidence that silencing GLI1 expression reduces the proliferation of U87-MG and T98G cells to abrogate disease progression. We also demonstrate that silencing GLI1 promotes sensitivity to TMZ by broadly reducing efflux behavior attributed to multidrug transporters. Further, we show that Hh pathway inhibition induces the expression of wild-type, but not mutant p53, suggesting that silencing GLI1 may induce tumor suppression via a p53-dependent mechanism. We initially hypothesized that GLI1 silencing without TMZ co-treatment would induce apoptosis via p53, however, we observed activation of separate tumor suppressive pathway. Specifically, we found that silencing GLI1 induces senescence rather than apoptosis, and this occurs via a mechanism that depends on the absence of PTEN. Finally, we demonstrate that combined Hh/GLI1 inhibition and TMZ treatment induces apoptosis and suppresses the growth of U87-MG cells cultured as neurospheres, suggesting an abrogation of glioma stem cell-like behavior. In aggregate, this data warrants the continued investigation of Hh-targeted therapies as adjuvants for GBM management.

## RESULTS

### U87-MG and T98G GBM cells exhibit active Hh signaling required for proliferation

**Figure 1:**
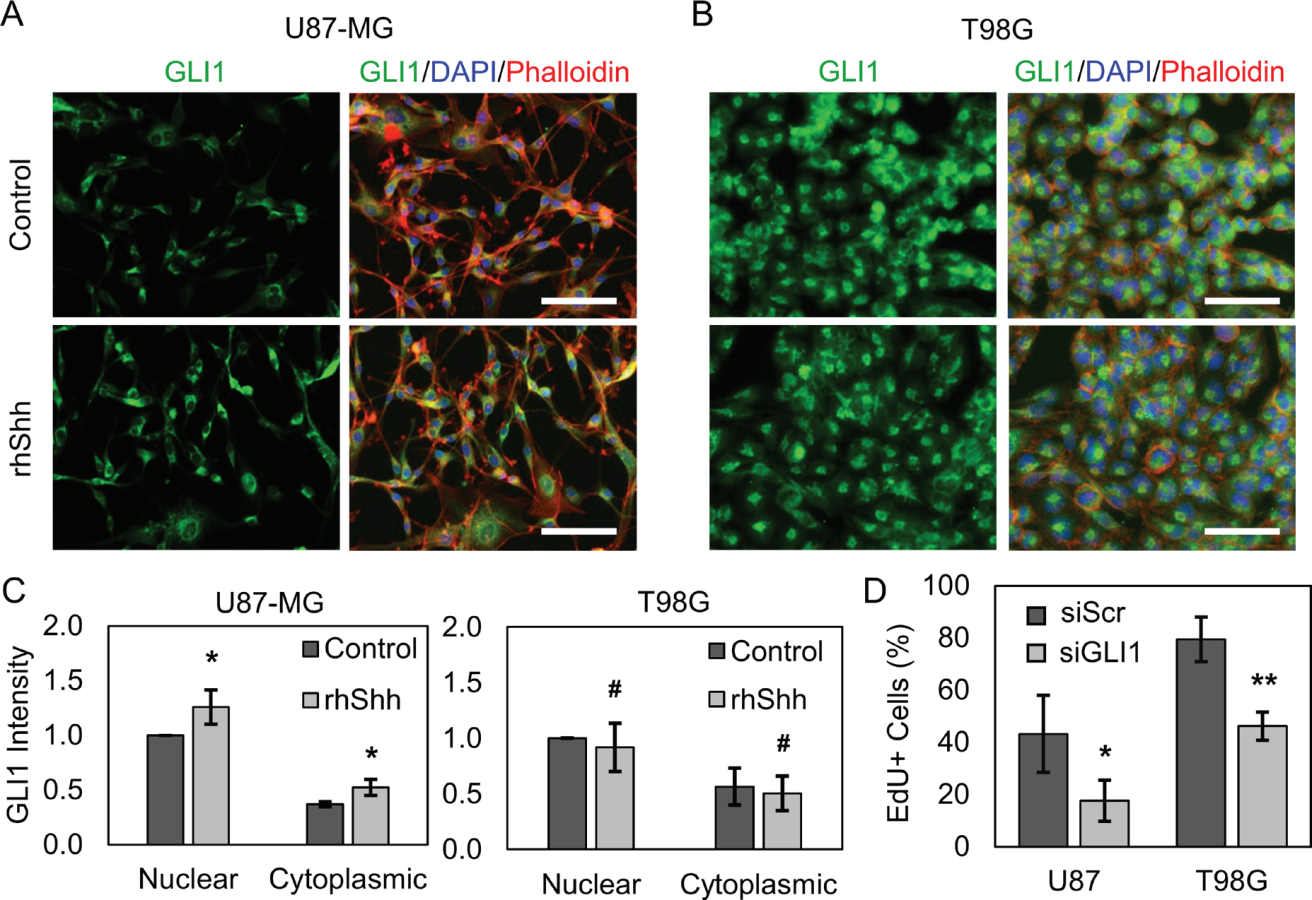
U87-MG and T98G GBM cells exhibit active Hh signaling via GLI1 rhShh increases GLI1 expression and nuclear translocation in (**A**) U87-MG but not (**B**) T98G GBM cells by immunofluorescence. Scale bars = 100 µm. (**C**) Quantitative image analysis reveals that U87-MG GLI1 intensity is significantly increased by ∼30% in the nucleus and by ∼40% in the cytoplasm relative to that in control cells. Data are shown as mean ± standard deviation from 3 independent experiments, ^*^p < 0.05 by Student’s *t*-test relative to control. Changes in T98G GLI1 intensity are insignificant. # no significance by Student’s *t*-test. (**D**) By EdU incorporation and flow cytometry analysis, silencing GLI1 decreases the proliferation of U87-MG and T98G cells by ∼60% and ∼44%, respectively, relative to siScr. Data are shown as mean ± standard deviation, ^*^p = 0.03, ^**^p = 0.002 by paired *t*-test relative to control.

In initial studies, we aimed to validate that both U87-MG and T98G cells exhibit active Hh signaling, making them suitable *in vitro* GBM models for this work. Nuclear localization of GLI1 was taken to indicate Hh pathway activation, as active Hh signaling produces GLI1 transcriptional activity and cytoplasmic GLI1 undergoes proteasomal degradation [33]. U87-MG and T98G cells were treated with recombinant human Shh (rhShh) for 48 hours and assessed for GLI1 expression using immunofluorescence. Images obtained using fluorescence microscopy reveal that GLI1 is present in both the nucleus and cytoplasm of untreated U87-MG and T98G cells, suggesting that Hh signaling is active in both cell lines. Further, stimulation with rhShh increases U87-MG GLI1 staining intensity by ∼30% in the nucleus and ∼40% in the cytoplasm (Figure 1A, 1C). In contrast, GLI1 staining intensity is conserved with rhShh treatment in T98G cells (Figure 1B, 1C), indicating that pathway activity is already maximal in untreated culture, that GLI1 is primarily regulated by other Hh ligands (Indian, Desert hedgehog), or by noncanonical signaling mechanisms in these cells.

GLI1 is well understood to positively regulate cell proliferation through direct transcriptional upregulation of cyclinD/E, Rb tumor suppressor inhibitors, c-Myc, and other pro-proliferative genes [34–36]. Therefore, we hypothesized that silencing GLI1 should decrease the proliferation of both U87-MG and T98G cells. To test this, U87-MG and T98G cells were transiently transfected with siRNA against GLI1 (siGLI1) or a scrambled control siRNA (siScr) and subsequently analyzed by an EdU incorporation assay. Flow cytometric analysis revealed that silencing GLI1 decreased the fraction of proliferating, EdU+ U87-MG cells by ∼60% and the fraction of EdU+ T98G cells by ∼44% (Figure 1D, Supplementary Figure 2). These results further support the presence of Hh pathway activity in U87-MG and T98G cells even without stimulation with exogenous rhShh.

### Silencing GLI1 potentiates GBM cell response to TMZ by decreasing multidrug efflux activity

**Figure 2:**
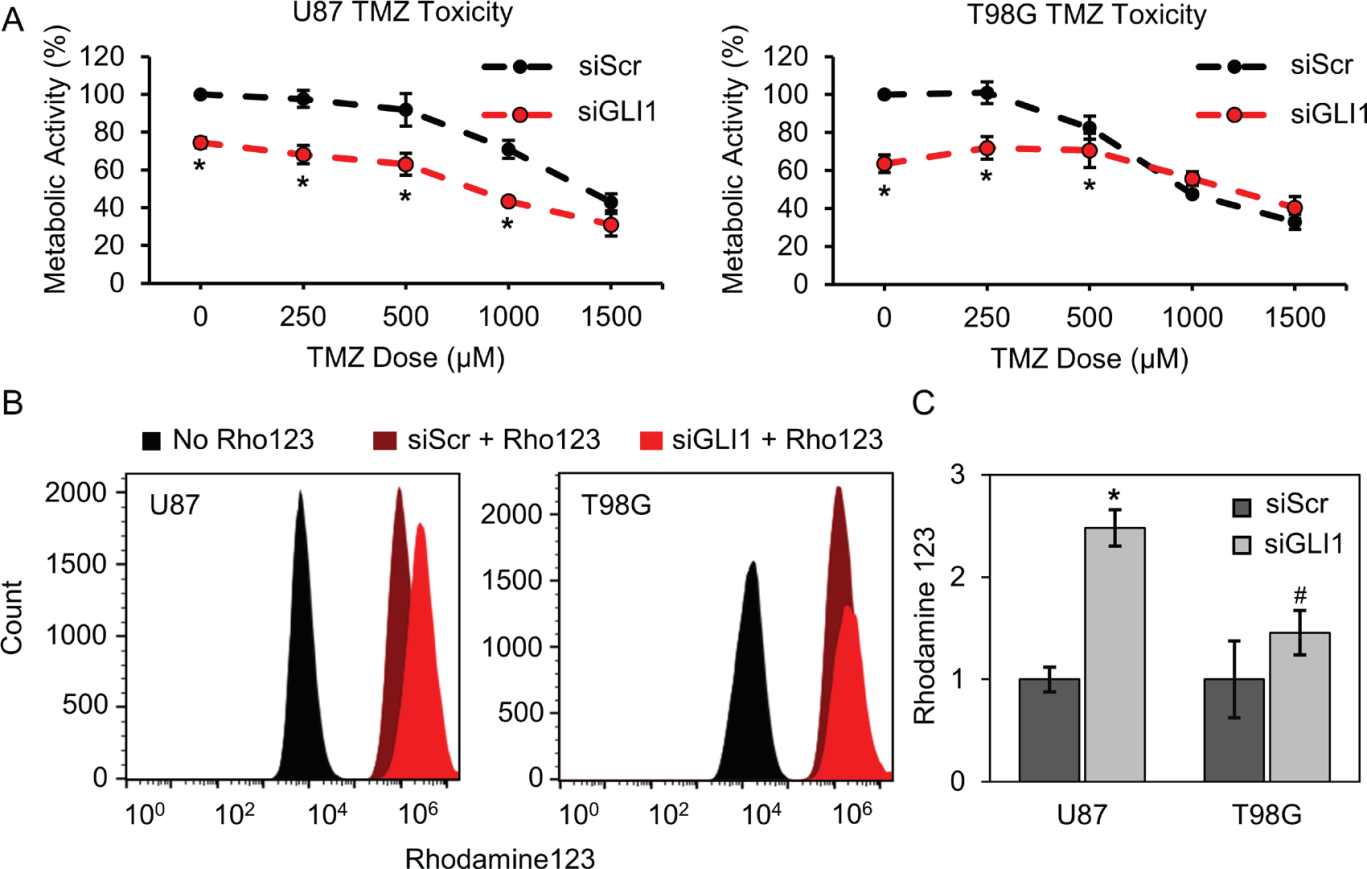
Silencing GLI1 influences GBM cell response to TMZ and reduces multidrug efflux activity (**A**) By AlamarBlue assay, U87-MG and T98G metabolic activity is significantly decreased with combined GLI1 silencing and low-dose TMZ treatment. Data are shown as mean ± standard deviation, ^*^p < 0.01 by one-way ANOVA with post-hoc Tukey. (**B**) Silencing GLI1 reduces multidrug efflux activity in both U87-MG and T98G cells. Flow cytometry reveals that silencing GLI1 prior to incubating cells with Rhodamine123 increases cellular Rhodamine123 intensity by 2.5-fold and 1.5-fold in U87-MG and T98G cells, respectively (**C)**. Data are shown as mean ± standard deviation, ^*^p = 0.0008, ^#^*p* = 0.07 by paired *t*-test relative to siScr.

Having established that Hh signaling is active in both of our GBM cell models and contributes to cell proliferation, we asked whether Hh/GLI1 activity contributes to cellular resistance to TMZ. To test this, U87-MG and T98G cells were transiently transfected with siRNA targeting GLI1 (siGLI1) or a scrambled control (siScr), then treated with a range of TMZ doses (0–1500 µM). Treatment efficacy was evaluated using an AlamarBlue assay to measure cellular metabolic activity. In U87-MG cells, we found that silencing GLI1 prior to TMZ treatment decreases metabolic activity up to 30% for TMZ doses through 1000 µM, past which we no longer observed increased efficacy with co-treatment (Figure 2A). We further conducted simple analysis to determine whether the treatments employed in tandem were synergistic, additive, or antagonistic. Briefly, the projected additive effect was calculated by multiplying the metabolic activity fraction for each treatment (siGLI1 or TMZ) individually. If the measured effect was greater than the projected additive, the co-treatment was considered synergistic. Conversely, if the measured effect was less than the projected additive, the co-treatment was considered antagonistic. Using this analysis method, we found that co-treating U87-MG cells with siGLI1 and TMZ produces an additive decrease in metabolic activity for all TMZ doses tested (Supplementary Figure 3). In T98G cells, while silencing GLI1 alone significantly reduced metabolic activity, co-treatment with TMZ induced an additive therapeutic effect at 250 µM TMZ, and an antagonistic therapeutic effect was exerted at higher TMZ doses (Figure 2A, Supplementary Figure 3). These results suggest that additional signaling mechanisms are important for regulating GBM cell response to TMZ, especially in T98G cells, but support that Hh inhibitors may improve the anticancer efficacy of low TMZ doses.

We further sought to delineate mechanisms by which silencing GLI1 may improve GBM cell TMZ response. Previous research has demonstrated that GLI1 transcriptionally regulates several transmembrane efflux transporters, such as MDR1/ABCB1, MRP1, LRP, and BCRP/ABCG2, which mediate chemotherapy resistance in several cancers [27, 37, 38]. Therefore, we asked whether silencing GLI1 could translate to a net reduction in GBM cell multidrug efflux activity. To test this, we used a dye-based efflux activity assay in which intracellular rhodamine intensity is taken to indicate small molecule retention. Rhodamine123 is a suitable dye choice for these experiments because it is a known substrate for multidrug efflux transporters [39]. In these experiments, U87-MG and T98G cells were transiently transfected with siGLI1 or siScr, then incubated with Rhodamine123 for 20 minutes. The dye was removed and cells were further incubated in fresh media for an additional 20 minutes. Cells were then trypsinized and analyzed for Rhodamine123 intensity by flow cytometry. Flow cytometry analysis reveals that silencing GLI1 can increase the intracellular Rhodamine123 intensity by 2.5-fold in U87-MG cells and 1.5-fold in T98G cells (Figure 2B, 2C). Therefore, we conclude that silencing GLI1 can reduce multidrug efflux activity to improve the retention of small molecule chemotherapeutics.

### Suppressing Hh signaling modulates p53 and MGMT expression in GBM cells

**Figure 3:**
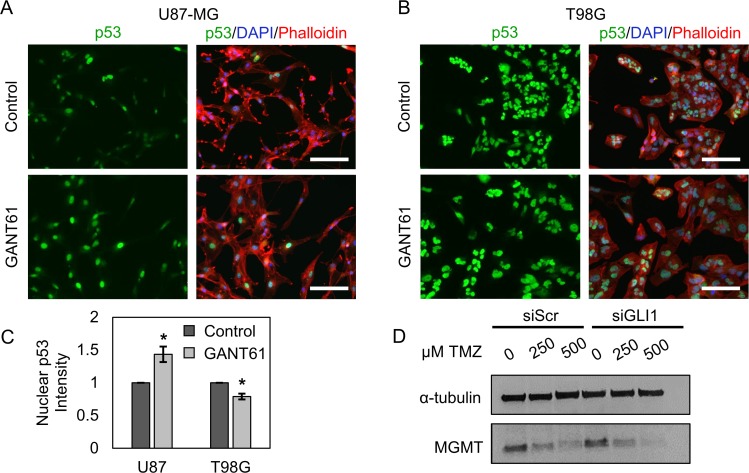
Hh inhibitors modulate p53 and MGMT expression in GBM cells Nuclear p53 staining intensity increases with GANT61-mediated Hh inhibition in (**A**, **C**) U87-MG cells by 43.4%, but decreases in (**B**, **C**) T98G cells by 21.5% relative to that in control cells. Data are shown as mean ± standard deviation from 3 independent experiments, ^*^p < 0.005 by Student’s *t*-test. Scale bars = 50 µm. (**D**) By Western blotting, T98G MGMT expression decreases in a TMZ-dependent manner. TMZ-induced downregulation of MGMT may be potentiated by GLI1 silencing.

Next, we asked whether Hh pathway inhibition could restore tumor suppressive activity in GBM cells. To evaluate tumor suppressive activity in U87-MG and T98G cells, we used immunofluorescent staining to assess the relative expression and localization of p53. Wild-type p53 transcriptionally regulates multiple genes involved in apoptosis, cell cycle arrest, DNA repair, and senescence in response to DNA damage such as that induced by hypoxia, radiation, or chemotherapy [28]. Thus, upregulation of wild-type p53 should induce tumor suppressive functions to mediate GBM cell death in response to chemotherapy. The role of mutant p53 in GBM is more poorly understood, and mutant p53 may promote both tumor suppressive and tumorigenic cell signaling. Both wild-type and mutant p53 are represented in this study, where U87-MG cells express wild-type p53 and T98G cells express a mutant p53 variant [9]. Cells seeded on coverslips were treated with the pharmacological GLI inhibitor GANT61 [40] for 48 hours, then stained for p53 and analyzed by quantitative fluorescence microscopy. We demonstrate that GANT61 significantly increases nuclear p53 by 43.4% in U87-MG cells (Figure 3A, 3C) and decreases nuclear p53 by 21.5% in T98G cells (Figure 3B, 3C).

We further investigated the potential role of MGMT in T98G resistance to TMZ, since MGMT is expressed highly in T98G cells. One previous study investigating other GBM cell lines reported that MGMT can be up-regulated in response to TMZ [7], so we asked whether TMZ-mediated upregulation of MGMT could be driving TMZ resistance in T98G cells. T98G cells were transfected with siGLI1 or siScr and subsequently treated with 250–500 µM TMZ for 48 hours, then lysed and assessed for MGMT expression by Western blotting. Our results show that MGMT expression is reduced in T98G cells treated with TMZ in a dose-dependent manner, and this MGMT downregulation may be enhanced when TMZ is combined with siGLI1 (Figure 3D). Accordingly, other mechanisms may be predominantly responsible for T98G TMZ resistance under Hh pathway inhibition.

### Silencing GLI1 does not induce apoptosis in GBM cells without TMZ co-treatment

**Figure 4:**
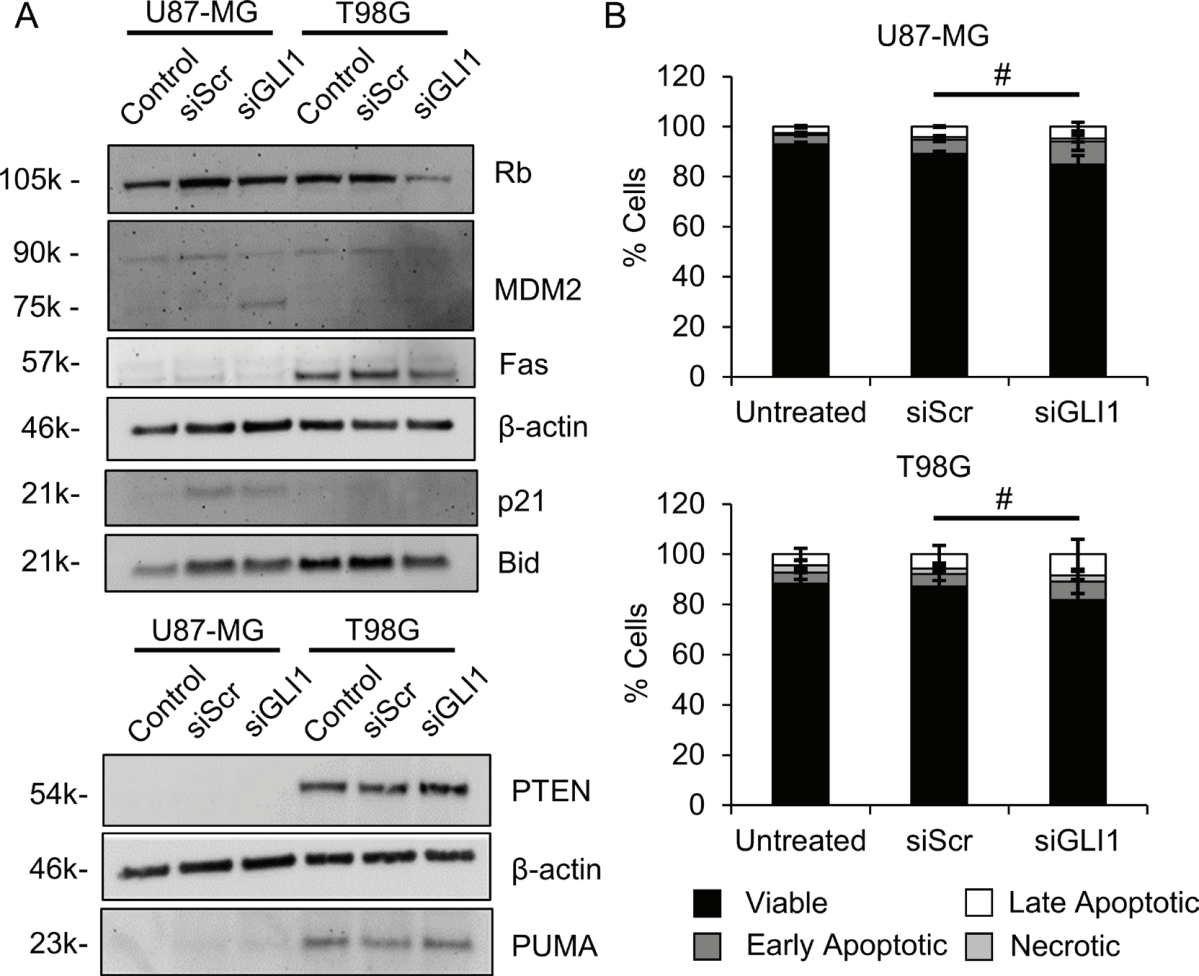
Silencing GLI1 does not correlate with apoptosis in GBM cells (**A**) By Western blotting, proteins associated with apoptosis induction do not increase with GLI1 silencing. (**B**) GLI1 silencing does not significantly increase AnnexinV-FITC/PI staining, # no significant difference in the fraction of viable, early apoptotic, late apoptotic, or necrotic cells by one-way ANOVA with posthoc Tukey.

Because Hh pathway suppression increased wt-p53 in U87-MG cells and decreased mut-p53 in T98G cells, we hypothesized that silencing GLI1 might induce apoptosis in GBM cells even in the absence of TMZ co-treatment. To test this, we first evaluated the expression of proteins downstream of p53 that might be upregulated in p53-mediated apoptosis. Contrary to our expectation, in U87-MG cells treated with siGLI1, we observed decreases in Rb, p21, Bid, and MDM2 expression (Figure 4A). In T98G cells, we observed slight increases in PTEN and PUMA expression and decreases in Rb, Fas, Bid, and MDM2 expression by Western blotting (Figure 4A). To validate these surprising results and ensure that p53-mediated apoptosis was not occurring by another signaling mechanism, we used AnnexinV-FITC/PI staining to directly assess apoptosis in cells treated under identical conditions. Surprisingly, we observed a slight but insignificant increase in the fraction of apoptotic cells (AnnexinV+, PI±) following GLI1 silencing, with 14 ± 3.6 % of cells treated with siGLI1 staining AnnexinV+ versus 9.9 ± 0.9% of cells treated with siScr (Figure 4B). Therefore, we concluded that direct induction of apoptosis by a p53-dependent mechanism is unlikely to cause the previously observed decreases in proliferation and metabolic activity with GLI1 silencing.

### Silencing GLI1 induces senescence in GBM cells in a manner dependent on the absence of PTEN

**Figure 5:**
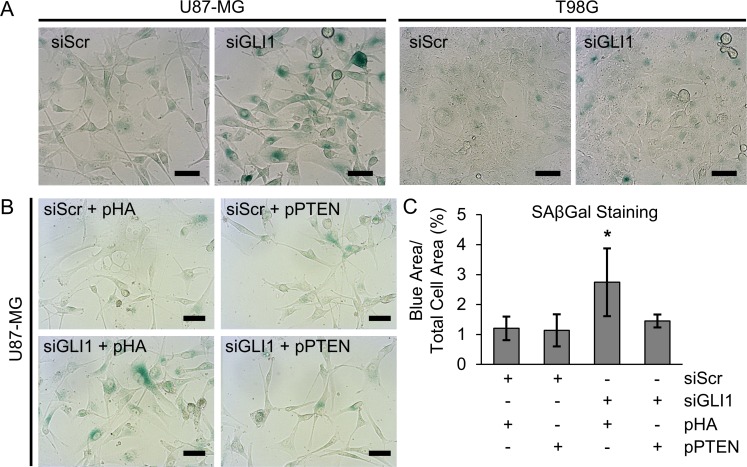
Silencing GLI1 induces senescence in U87-MG cells in a manner dependent on the absence of PTEN (**A**) SAβGal staining (teal) demonstrates that silencing GLI1 induces senescence in U87-MG cells. (**C**) Quantitative image analysis across three independent experiments confirms that (**B**) PTEN expression reverses siGLI1-induced senescence, ^*^p < .05 by one-way ANOVA with post-hoc Tukey test.

Our studies produced the surprising result that although silencing GLI1 reduces GBM cellular metabolic activity alone and in combination with TMZ and modulates p53 expression, this does not significantly induce apoptosis in the absence of TMZ co-treatment. Therefore, we asked whether we could be observing senescence rather than apoptosis in response to siGLI1 alone. To test this, we used senescence associated β-galactosidase (SAβGal) staining to identify senescent cells following GLI1 silencing. We observed a marked increase in SAβGal staining (Figure 5A) in U87-MG cells treated with siGLI1, but not in T98G cells. Interestingly, previous research has identified that loss of PTEN can induce premature senescence, which may have a compensatory role for apoptosis in this context [41]. Because U87-MG cells exhibit loss of PTEN while T98G cells do not (Figure 4A), we hypothesized that loss of PTEN might explain the observed senescent phenotype in response to siGLI1. Indeed, we observed that inducing PTEN expression reversed this behavior; co-transfecting U87-MG cells with siGLI1 and a control plasmid (pHA) significantly increased SAβGal staining by 2.3-fold relative to cells co-transfected with siScr and pHA, while co-transfection with siGLI1 and pPTEN reversed this phenotype (Figure 5B, 5C). Therefore, we concluded that silencing GLI1 induces senescence in a manner dependent on the absence of PTEN.

### Hh inhibition and TMZ co-treatment promotes apoptosis in neurospheres and impairs neurosphere growth

**Figure 6:**
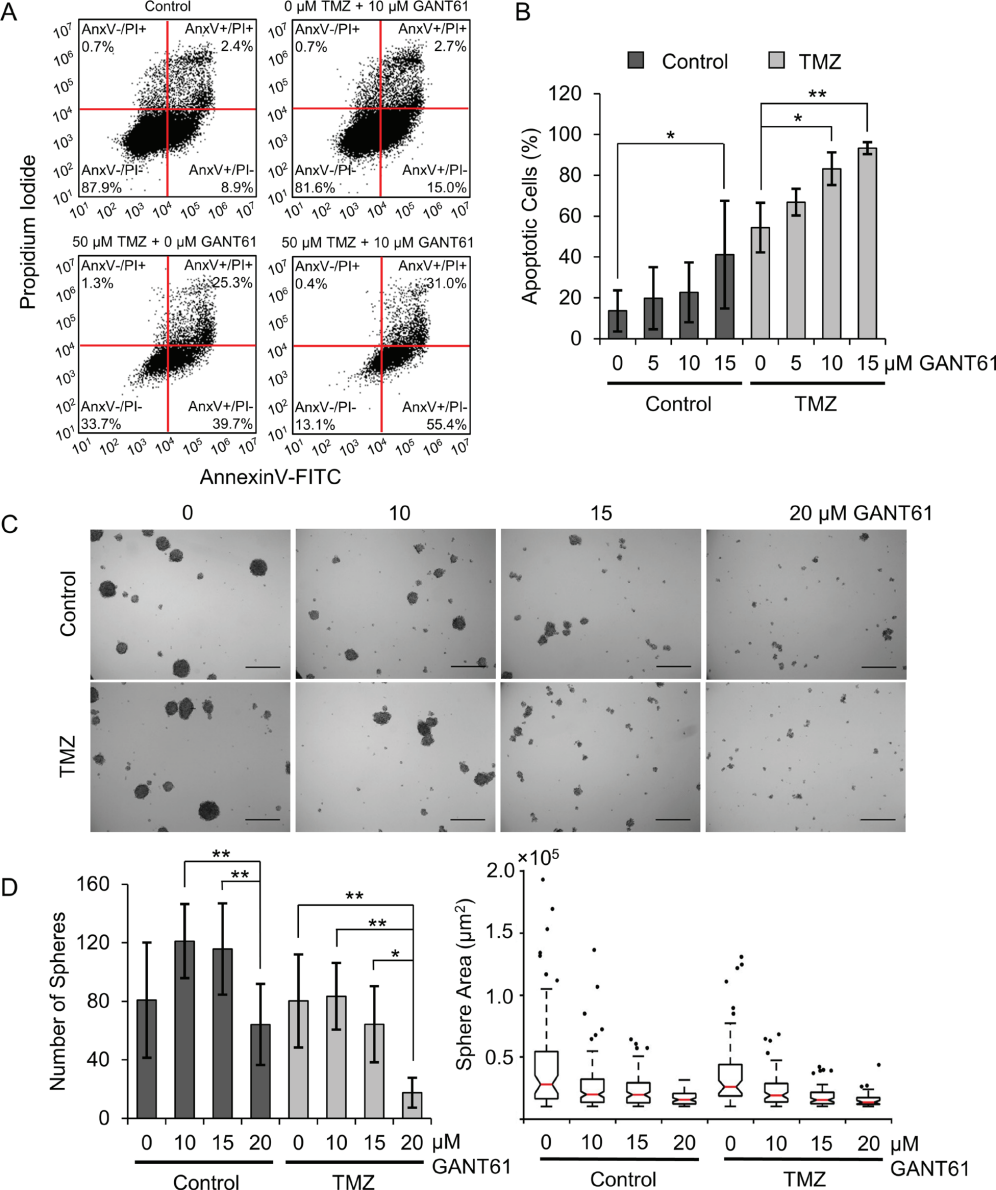
Hh inhibitors and TMZ cooperatively promote apoptosis in U87-MG neurospheres and suppress neurosphere growth (**A**) Flow cytometric scatterplots displaying AnnexinV-FITC and PI staining intensities in U87-MG cells grown as neurospheres in medium containing GANT61 (0 or 10 µM) and TMZ (0 or 50 µM TMZ) from one representative experiment. (**B**) The fraction of apoptotic (AnnexinV+) U87-MG cells grown as neurospheres in medium containing GANT61 and TMZ (50 µM TMZ), summarized across 3 independent experiments. Data shown are means ± standard deviation from 3 independent experiments, ^*^p < 0.05, ^**^p < 0.01 by one-way ANOVA with post-hoc Fisher’s least significant difference test. (**C**) Representative brightfield images of U87-MG neurospheres grown for one week in medium containing GANT61 and TMZ (10 µM TMZ). Scale bar = 500 µm. (**D**) *(Left)* Number of spheres counted by automated image analysis, averaged across 9 independent replicates. Data shown are means ± standard deviation from 9 independent experiments, ^*^p < 0.05, ^**^p < 0.01 by one-way ANOVA with post-hoc Tukey test. *(Right)* Sphere size (projected area) as determined by automated image analysis from one representative experiment.

Having established that Hh inhibition induces senescence in a loss of PTEN-dependent manner as a standalone therapy, we were interested in the fate of GSCs co-treated with Hh inhibitors and TMZ. To model this, we used an *in vitro* model of neurosphere formation in which neurospheres were grown from a single cell suspension in serum-free, chemically-defined medium. Importantly, GBM cells grown as neurospheres are more sensitive to clinically-relevant TMZ doses than GBM cells grown in adherent culture [42], so we anticipated that neurospheres would be a more appropriate model to elucidate the effects of Hh inhibition and TMZ co-treatment. We validated that U87-MG cells grown as neurospheres are more stem-like than adherent cells using qPCR to measure changes in the expression of genes associated with this phenotype (Supplementary Figure 4). We found that U87-MG neurospheres exhibit a 10-fold increase in CD133 expression and a 5-fold increase in Nanog expression (Supplementary Figure 4). Next, we investigated the extent of apoptosis induced in U87-MG GBM neurospheres by co-treatment with GANT61 and TMZ using AnnexinV/PI staining. We found that, as a monotherapy, only the highest tested GANT61 dose (15 µM) is sufficient to induce a significant increase in apoptotic (AnnexinV+, PI±) cells relative to untreated controls (Figure 6A, 6B, Supplementary Figure 5), which increased the fraction of apoptotic cells to 41%. While TMZ alone induces apoptosis in 54% of cells, the addition of 10 or 15 µM GANT61 significantly increases the fraction of apoptotic (AnnexinV+, PI±) cells to 83% or 93%, respectively (Figure 6A, 6B, Supplementary Figure 5). Further, synergy analysis using the previously described method revealed that co-treating U87-MG neurospheres with TMZ and 10 or 15 µM GANT61 produced a statistically significant synergistic decrease in the fraction of viable (AnnexinV-, PI-) cells (Supplementary Figure 6). This data supports the hypothesis that co-treatment with TMZ and GANT61 may cooperatively promote apoptosis in GBM neurospheres. We next investigated the consequences of GANT61/TMZ-mediated apoptosis on neurosphere growth. Using brightfield microscopy, we observed a striking decrease in neurosphere size induced by GANT61/TMZ co-treatment and correlating with increased doses of GANT61 (Figure 6C, 6D; Supplementary Figure 7). Quantitative image analysis also revealed that GANT61 significantly reduced the number of neurospheres formed with or without co-treatment with TMZ (Figure 6D).

## DISCUSSION

In this study, we evaluated the therapeutic potential of Hh pathway inhibition as an adjuvant to TMZ chemotherapy using *in vitro* models of GBM. Through this work, we sought to identify key molecular and phenotypic changes that occur in GBM cells because of co-treatment with Hh pathway inhibitors and TMZ. We found that silencing GLI1 before administering TMZ to GBM cells enhanced the cytotoxic effects of chemotherapy, and this effect is likely dependent on the status of additional GBM-promoting genes. Further, this enhanced TMZ cytotoxicity correlated with a decrease in multidrug efflux activity and an increase in wild-type p53, a key mediator of apoptosis, cell cycle arrest, and senescence [28]. Interestingly, the increase in p53 observed in U87-MG cells did not induce apoptosis, but instead triggered senescence in a manner dependent on the loss of PTEN expression. Finally, we found that co-treating GBM cells in neurosphere culture with Hh pathway inhibitors and TMZ promotes apoptosis and reduces neurosphere formation, suggesting an inhibition of glioma stem cell-like behavior. Overall, this work provides evidence that Hh pathway inhibition may overcome cellular mechanisms that promote TMZ resistance, and the data warrant continued investigation of this combination treatment.

Previous research has demonstrated that Hh inhibition through gene regulatory or pharmacological means can sensitize GBM cells to TMZ [18, 23, 43] and has suggested a number of possible mechanisms to support these findings. Although many researchers either do not observe MGMT expression in U87-MG cells [9, 30, 31] or observe very little MGMT expression [44, 45], one study reported that GANT61-mediated Hh pathway suppression improved TMZ sensitivity by repressing MGMT and Notch proteins in U87 and U251 cells [43]. Consistently, another investigation reported that targeting both Hh and Notch signaling could further improve TMZ sensitivity in GBM cells [18]. In the present study, we demonstrate that silencing GLI1 prior to TMZ treatment can additively improve chemotherapeutic efficacy in U87-MG cells cultured in adherent conditions (Figure 2A). Notably, U87-MG cells are highly refractory to TMZ treatment in adherent culture. This is evident in that minimal U87-MG toxicity is observed for TMZ doses up to 500 µM, approximately 10-fold higher than the maximum clinically feasible TMZ dose [46]. Accordingly, it is encouraging that the therapeutic benefit afforded by co-treatment was most prominent for lower TMZ doses, suggesting that the combination of Hh inhibitors and low-dose TMZ may improve treatment outcomes while mitigating off-target toxicity. We can extend these findings to U87-MG cells grown in neurosphere culture, thought to be more representative of a GSC-rich population. Interestingly, we observed a synergistic, or greater than additive, therapeutic effect induced by co-treatment with GANT61 and TMZ in these models (Figure 6A, 6B, Supplementary Figure 6). We suspect this is because Hh signaling is upregulated in GSCs relative to differentiated GBM cells [18], and neurospheres may consequently be more sensitive to its suppression. Additionally, some GBM cell lines grown as neurospheres exhibit greater sensitivity to chemotherapeutic agents including carmustine and TMZ than GBM cell lines grown in adherent culture [42]. Surprisingly, and in contrast to our observations in U87-MG cells, our studies show that silencing GLI1 prior to treating T98G cells with TMZ chemotherapy at doses greater than 250 µM produced an antagonistic therapeutic effect (Figure 2A, Supplementary Figure 3). Though we initially hypothesized that this could be due to MGMT upregulation, we found that siGLI1/TMZ co-treatment decreases MGMT expression at TMZ doses greater than 250 µM (Figure 3D), and we concluded that other mechanisms are responsible for this antagonistic therapeutic effect. We speculate that this may occur because silencing GLI1 may relieve GLI1-mediated suppression of mutant p53, which can oppose TMZ cytotoxicity [10, 28]. These studies warrant future investigation to delineate the cause of this differential drug response.

Further, Hh signaling has been linked to the upregulation of drug efflux transporters that reduce the intracellular concentration of small molecule chemotherapeutics, rendering them ineffective and imparting drug resistance [27, 37, 38, 47]. While several investigations have shown that Hh inhibition decreases the expression of one or more such transporters at the protein level, we demonstrate here that silencing GLI1 does produce a net decrease in multidrug efflux activity. In these studies, we used Rhodamine123 as a tracer dye to indicate efflux activity, as Rhodamine123 is a known substrate for such transporters. Our data show that silencing GLI1 prior to incubating U87-MG cells with Rhodamine123 can significantly improve intracellular dye intensity, and therefore retention, by ∼2.5-fold. T98G cells exhibit a slight but statistically insignificant increase in Rhodamine123 retention (Figure 2B, 2C). These results are consistent with our chemosensitization studies, which revealed that silencing GLI1 increases TMZ response in U87-MG cells to a much greater degree than in T98G cells.

It is also of great importance to consider the impact Hh inhibition might have on signaling pathways with well-established significance in TMZ resistance. Both wild-type and mutant p53 have been implicated in the progression and therapeutic response of GBM tumors. Wild-type p53 is well known to mediate apoptosis, cell cycle arrest, and senescence in response to genotoxic stress through a complex tumor suppressive signaling network. Despite the presence of altered p53 in 25–30% of primary GBM cases [28], much remains unknown regarding the phenotypic consequences of p53 mutants on GBM progression. Previous research has identified a GLI1-p53 negative feedback loop present in neural stem cells and brain tumor cells, where GLI1 knockdown was found to increase both p53 and active phospho-serine15 p53 in U87-MG cells [48]. However, additional signaling mechanisms also regulate the inverse relationship between GLI1 and p53. Upstream of GLI1, constitutively activated Smoothened mutants upregulate MDM2, which then represses p53 tumor suppression activity [49]. A Hh-p53 negative regulatory loop is further maintained due to competitive GLI1 and p53 binding to the coactivator TATA Binding Protein Associated Factor 9 (TAF9) [50] and Nanog-mediated upregulation of GLI1 and downregulation of p53 [51]. Consistent with these prior findings, we have demonstrated that pharmacological GLI inhibition can increase p53 expression U87-MG cells. However, we also report the surprising result that this relationship is not conserved in T98G cells (Figure 3A–3C).

Because Hh pathway suppression increased wt-p53 in U87-MG cells and decreased mut-p53 in T98G cells, we hypothesized that silencing GLI1 might induce apoptosis in GBM cells, even in the absence of TMZ co-treatment. To test this, we assessed the expression of proteins downstream of p53 known to mediate apoptosis. Surprisingly, we did not find increased expression of proteins involved in p53-mediated apoptosis (Figure 4A), nor did we observe significant increases in AnnexinV-FITC/PI staining with GLI1 silencing (Figure 4B). This unexpected result led us to consider senescence as an alternative explanation for the observed decreases in proliferation and metabolic activity with siGLI1 alone. Interestingly, we did find that silencing GLI1 induced a senescent phenotype in U87-MG cells but not in T98G (Figure 5A). While silencing GLI1 and GLI2 has recently been demonstrated to induce senescence to restore chemosensitivity in melanoma cells [52], Hh signaling has not yet been linked to senescence in GBM. Thus, our results provide novel insight into Hh signaling as a means of evading senescence in GBM cells. Further, previous research has identified that loss of PTEN can induce premature senescence, which may have a compensatory role for apoptosis in this context [41]. Here, we demonstrate that while siGLI1 induces senescence, expressing PTEN simultaneously with GLI1 silencing can prevent this phenotype (Figure 5B). While p53 and loss of PTEN have both been implicated in glioma cell senescence, we report the novel finding that senescence can also be induced by Hh pathway suppression in the absence of PTEN. While much remains unknown about the role of senescence in tumor progression, senescence is thought to suppress tumor growth in that senescent cells no longer exhibit limitless replicative potential [53]. However, senescent cells that have incurred DNA damage also acquire a senescence-associated secretory phenotype (SASP), in which senescent cells secrete pro-inflammatory factors that can promote tumor progression [54]; this warrants future research on the effects of siGLI1-induced SASP on GBM progression.

Also heavily implicated in TMZ resistance, MGMT is a DNA repair enzyme that removes alkyl groups transferred to the O^6^ position of guanine by TMZ to oppose drug toxicity. Though MGMT expression is primarily regulated epigenetically [8], a GLI1-binding domain has been identified within the MGMT promoter in medulloblastoma [32], and Hh inhibition has been linked to MGMT downregulation in GBM cell lines [27, 43]. Further, additional reports have sought to relate MGMT expression to TMZ treatment [7, 44, 55], though no consensus has been reached on whether TMZ modulates MGMT expression. For example, one study reported that exposing GBM cell lines to TMZ in a cyclic manner (3 days of treatment followed by 3 days without drug, repeated twice) resulted in MGMT protein upregulation in SF268 GBM and SK-N-SH neuroblastoma cell lines [7]. Another recent report demonstrated that MGMT protein levels are not altered following 72 hours of 10 µM TMZ exposure in T98G GBM cells [44]. In the present study, we observed a dose-dependent decrease in MGMT expression by T98G cells following treatment with 250–500 µM TMZ for 48 hours (Figure 3D). There are numerous possible explanations for this discrepancy, as MGMT is regulated by several factors, including mutant p53 [9], MEK-ERK [56], mTOR [44], hypoxia [15, 57], and microRNAs [58, 59]. Further, the TMZ dose-dependent MGMT decrease we observed is enhanced with GLI1 silencing (Figure 3D). Our data indicate that TMZ treatment and GLI1 silencing cooperatively deplete MGMT expression, which is consistent with previous reports [27, 43]. Our studies, along with previous research, highlight the importance of investigating combination therapies in multiple GBM models to better understand differential response to treatment and predict efficacious therapy combinations.

Having established that Hh inhibition induces senescence in a loss of PTEN-dependent manner as a standalone therapy, we were interested in the fate of GBM neurospheres co-treated with Hh inhibitors and TMZ. Our studies revealed that pharmacological Hh inhibition with GANT61 in combination with TMZ can induce apoptosis in U87-MG neurospheres, which consequently reduces both neurosphere size and the number of neurospheres formed (Figure 6A–6D, Supplementary Figures 5–7). Interestingly, our studies reveal that while TMZ as a monotherapy reduces the number of large spheres (imaged area >1 × 10^5^ µm^2^) with no significant change in the total number of spheres, the addition of GANT61 does significantly reduce the sphere count. This is supported by previous work, which shows that pharmacological or genetic Hh pathway suppression, but not TMZ alone, can prevent GSC tumorigenicity in mice [23], and Hh and Notch pathway inhibition enhances TMZ sensitivity in CD133+ stem-like cells [18]. Our results demonstrate that GANT61/TMZ co-treatment suppresses the growth of anchorage-independent U87-MG cells thought to be more representative of a GSC-rich subpopulation. Taken together with our previous results, this suggests that senescence mediated by siGLI1 monotherapy is indeed tumor suppressive and potentiates the effects of TMZ chemotherapy.

Our studies indicate that Hh pathway suppression may enhance the cytotoxic effects of TMZ against GBM, particularly those expressing low levels of MGMT and wild-type p53. We demonstrated that Hh inhibition reduces multidrug efflux activity, modulates p53 and MGMT expression, induces senescence dependent on the absence of PTEN, and reduces the growth of glioma cells exhibiting a stem-like phenotype. Overall, our data highlight the importance of investigating how Hh-targeted therapies interact with pathways that promote cellular resistance mechanisms to ultimately achieve maximal tumor reduction and prevent recurrence.

## MATERIALS AND METHODS

### Cell culture and transient transfections

U87-MG and T98G cells were purchased from American Type Culture Collection (ATCC, Manassas, VA) and cultured in Dulbecco’s Modified Eagle Medium (DMEM) supplemented with 10% fetal bovine serum (FBS). Cells were maintained in a humidified incubator at 37° C, 5% CO_2_. RNA interference was performed using Dharmafect 1 (Dharmacon, Lafayette, CO) according to the manufacturer’s protocol to deliver 100 nM siRNA (Integrated DNA Technologies, Coralville, IA) to cells at approximately 50–60% confluence. Four hours post-transfection, media containing transfection reagents was removed and replaced with fresh media, and cells were incubated at 37° C, 5% CO_2_ for 72 hours prior to continued experimentation. siRNA sequences used in this study are as follows (written 5′ to 3′): siGLI1 sense: CCA GGA AUU UGA CUC CCA ATT, siGLI1 antisense: UUG GGA GUC AAA UUC CUG GCT, siScramble (siScr) sense: GUG CAC CAA CGA CUU AUC ATT, siScr antisense: UGA UAA GUC GUU GGU GCA CT. Plasmid DNA transfections were performed using Mirus TransIT X2 (Mirus Bio LLC, Madison, WI) to deliver 25 ng/mL pcDNA3-FLAG PTEN (Addgene #78777) or pcDNA3-FLAG HA (Addgene #10792) to cells at approximately 80% confluence. Twenty-four hours post-transfection, media containing transfection reagents was removed and replaced with fresh media, and cells were incubated at 37° C, 5% CO_2_ for 48 hours prior to analysis.

### Immunofluorescent staining and image analysis

Cells were seeded on coverslips in 24-well plates and grown overnight prior to treatment with either Hh activating agents or Hh inhibitors. For Hh activation experiments, cells were treated with recombinant human sonic hedgehog (rhShh) (R&D Systems, Minneapolis, MN) at 25 ng/ml to induce Hedgehog signaling and were incubated 48 hours at 37° C, 5% CO_2_. For chemical Hh inhibition experiments, cells were treated with 10 µM GANT61 (Cayman Chemical Company, Ann Arbor, MI) and were incubated 48 hours at 37° C, 5% CO_2_. Subsequently, coverslips were washed in phosphate buffered saline (PBS), fixed in ice-cold acetone (for GLI1 staining) or 4% formaldehyde (for p53 staining), and blocked in PBS containing 1% bovine serum albumin (BSA), 0.2% cold-fish gelatin, and 0.1% Tween-20 for 1 hour at room temperature. Coverslips were then probed with antibodies under the following conditions: 1:500 rabbit anti-GLI1 (Proteintech, Rosemont, IL) or 1:500 mouse anti-p53 (Santa Cruz Biotechnology, Santa Cruz, CA) diluted in blocking buffer at 4° C overnight, followed by incubation with AlexaFluor 488-conjugated secondary antibodies diluted in blocking buffer (1:1000, ThermoFisher Scientific, Waltham, MA) for 1 hour at room temperature. Cells were rinsed in PBS supplemented with 0.1% Tween-20, counterstained with 4’,6-diamidino-2-phenylindole (DAPI) to identify nuclei and phalloidin to identify actin cytoskeleton (Cell Signaling Technology, Danvers, MA), mounted on slides using gelvatol, and imaged on a Zeiss AxioObserver.Z1 microscope (Zeiss, Thornwood, NY). For each condition, 25 images were acquired from the central region of the coverslip using automated X, Y, and focus positioning.

Each image set was checked by a masked observer for out of focus images, which were discarded from analysis, resulting in sets of 22–25 images encompassing a minimum of 1000 cells per set. Each image was analyzed using a custom image analysis script in MATLAB (2016a; Mathworks, Natick, MA). Briefly, the image was segmented into nuclear and cytoplasmic regions and mean staining intensity was determined for each region. To identify the nuclear compartment, the DAPI stain was normalized and thresholded. To identify the cytoplasmic compartment, the phalloidin stain was normalized, morphologically closed with a 6 µm disk element, and thresholded. The thresholded stains were used as masks to determine the mean intensity of GLI1 and p53 staining in the two components. Data was analyzed frame by frame and averaged within each set.

### Western blotting

Protein samples were prepared by lysing cells in radioimmunoprecipitation assay (RIPA) buffer (Amresco, Solon, OH) supplemented with 2X Halt Protease and Phosphatase Inhibitor Cocktail (ThermoFisher Scientific, Waltham, MA) on ice. Sample protein concentration was measured using a detergent-compatible modified Lowry assay (Bio-Rad, Hercules, CA) relative to a BSA standard. Lysate was denatured in Laemmli buffer (Amresco, Solon, OH) at 99° C for 20 minutes, and 30 µg protein was loaded per well in a Bolt^®^ 4–12% Bis-Tris gel (ThermoFisher Scientific, Waltham, MA) and separated by electrophoresis at 135V for 1 hour. Protein was transferred to a 0.45 µm nitrocellulose membrane, which was subsequently blocked in 5% nonfat milk in tris-buffered saline containing 0.1% Tween-20 (TBST). Membranes were probed with primary antibodies at 4° C overnight, followed by incubation with anti-rabbit (1:25000) or anti-mouse (1:25000) horseradish peroxidase-conjugated secondary antibodies (Kirkegaard & Perry Laboratories, Inc., Gaithersburg, MD) for 1 hour at room temperature. Primary antibodies were obtained from Cell Signaling Technology (Danvers, MA) (MGMT, α-tubulin, β-actin) or Santa Cruz Biotechnology (Rb, p21, PTEN, PUMA, Bid, MDM2). Protein bands were detected by chemiluminescence using VisiGlo^™^ Select Chemiluminescent Substrate (Amresco, Solon, OH) and imaged on a ChemiDoc-It^2^ Imager (UVP, Upland, CA).

### Senescence analysis

Cells were seeded in 24-well plates at a density of 25,000 cells/well and cultured overnight prior to transfection with siGLI1 or pPTEN (or relevant controls). Cellular senescence was analyzed using a Senescence Associated β-Galactosidase (SAβGal) kit (Cell Signaling Technology, Danvers, MA) according to the manufacturer’s protocol. Stained cells were imaged using a Zeiss Axioobserver.Z1 microscope equipped with a color camera. For each condition, 25 images were acquired from the central region of the well plate using automated X, Y, and focus positioning. SAβGal staining was quantified using a custom MATLAB script. Briefly, cells were segmented from brightfield images using the Sobel method. RGB images were converted to HSV colorspace, and positive SAβGal staining regions within cells were thresholded using hue values from 0.265–0.58. The total area of SAβGal-positive regions was divided by the total area occupied by cells to obtain the fraction of SAβGal-positive area for each condition.

### Dose response, viability assay, and synergy assessment

GBM cytoxicity induced by temozolomide (TMZ, reconstituted in DMSO; Sigma-Aldrich, St. Louis, MO) chemotherapy was investigated following Hedgehog inhibition by silencing GLI1. Cells were seeded in 96-well plates at 5000 cells/well and transfected with siGLI1 (or siScr) as described above. Subsequently, cells were treated with increasing TMZ dosages (0–1500 µM) for 48 hours, and viability was assessed using an AlamarBlue assay (ThermoFisher Scientific, Waltham, MA) according to the manufacturer’s protocol with a 2-hour AlamarBlue incubation. Fluorescence intensity (Ex 550 nm/Em 585 nm) was recorded using a Synergy H1 Plate Reader (BioTek, Winooski, VT). Cells transfected with siScr and receiving no TMZ were taken to exhibit 100% metabolic activity, and cells treated with DMSO equivolume to the highest TMZ dosage diluted in media were used to verify that toxicity was due to TMZ treatment rather than DMSO-induced membrane permeabilization, since TMZ was reconstituted in DMSO prior to dilution in media.

Therapeutic synergy between GLI1 silencing and TMZ was assessed using previously reported methods [60]. For each group co-treated with siGLI1 and TMZ, a projected additive effect was calculated by multiplying the fraction of metabolic activity reduced by GLI1 silencing alone by the fraction of metabolic activity reduced by each TMZ dose. Then, the observed metabolic activities with co-treatment were compared to the projected values by one-way ANOVA with post-hoc Tukey. Co-treatments producing metabolic activities statistically greater than the projected value were taken to be antagonistic, statistically insignificant from the projected value were taken to be additive, and statistically lower than the projected value were taken to be synergistic.

### Assessment of drug efflux transporter activity

Rhodamine123 (Sigma-Aldrich, St. Louis, MO) efflux was measured to evaluate the impact of Hh/GLI1 inhibition on multidrug resistance (MDR) efflux transporter activity because it is a known substrate for membrane efflux transporters and its fluorescence is easily detectable [39, 60]. Cells were seeded in 24-well plates and grown overnight, then transfected with siGLI1 or siScr as described previously. Transfected cells were subsequently incubated with 5 µM Rhodamine123 for 20 minutes at 37° C, 5% CO_2_, then the Rhodamine123-containing medium was removed, cells were replenished with fresh media and incubated for another 20 minutes at 37° C, 5% CO_2_ to allow for efflux. Treated cells were harvested by trypsinization, fixed in 4% formaldehyde, resuspended in PBS and analyzed for Rhodamine123 fluorescence (Ex 488 nm/Em 533/30 nm) by flow cytometry using a BD Accuri™ C6 cytometer (Becton Dickinson Biosciences, Franklin Lakes, NJ).

### Proliferation assay

Cellular proliferation following Hedgehog inhibition by GLI1 silencing was evaluated using a Click-iT^®^ EdU Proliferation Assay (ThermoFisher Scientific, Waltham, MA). Cells seeded in 24-wells were transfected with siRNA as described previously and then incubated with 10 µM EdU for 16 hours at 37° C, 5% CO_2_. Cells were then harvested by trypsinization, washed in 1% BSA in PBS, fixed in 4% formaldehyde, permeabilized with 0.05% saponin, and stained according to the manufacturer’s protocol. EdU incorporation was measured by flow cytometry (Ex 488 nm/Em 533/30 nm) using a BD Accuri™ C6 cytometer (Becton Dickinson Biosciences, Franklin Lakes, NJ).

### Neurosphere growth and apoptosis analysis

Neurospheres were grown from a single-cell suspension of U87-MG’s in NeuroCult NS-A (STEMCELL Technologies, Vancouver, BC, Canada) medium supplemented with recombinant human epidermal growth factor (EGF, 20 ng/mL), recombinant human basic fibroblast growth factor (bFGF, 10 ng/mL), and heparin sulfate (2 µg/mL). Cells were plated in ultra-low adhesion 24-well plates at a density of 10,000 cells/mL for growth analysis in medium containing 10 µM TMZ and 10, 15, or 20 µM GANT61 for one week at 37° C, 5% CO_2_. Controls contained DMSO and/or ethanol (which are used to reconstitute TMZ and GANT61, respectively) at volumes equivalent to the highest TMZ and/or GANT61 doses used in the experiment. After one week, neurospheres were imaged for size and sphere forming efficiency analysis. Imaging was conducted on a Zeiss AxioObserver.Z1 microscope using automated stage control to image each well in its entirety. Following acquisition, images were stitched using Zeiss Efficient Navigation software (ZEN 2.0; Zeiss) and exported as single field images on the OME-TIFF standard and analyzed using a custom MATLAB script. Briefly, the well exterior was discarded by mapping an active contour to the high gradient region at the well edge [61]. After discarding the well exterior, varying background illumination was corrected using morphological reconstruction-based top-hat and bottom-hat filters [62] followed by homomorphic filtering. Image contrast was globally enhanced using homomorphic filtering and phase-preserving dynamic range compression and locally enhanced using the CLAHE algorithm [63, 64]. Following enhancement, spheres were identified as increases in local image entropy and touching spheres were separated using watershed-based segmentation. Automated sphere identification was manually confirmed for each image analyzed. Sphere growth data were reported as the average number of spheres per treatment group across 9 independent replicates, where spheres were defined as objects with diameter greater than 50 µm and eccentricity less than 0.8, and as size (sphere projected area) distributions for each treatment group. To evaluate apoptosis in U87-MG neurospheres treated with TMZ ± GANT61, neurospheres grown from a single-cell suspension at a density of 25,000 cells/mL were harvested after 6 days growth in NeuroCult NS-A medium ± 50 µM TMZ ± 0- 15 µM GANT61, trypsinized to a single-cell suspension, and analyzed using an AnnexinV-FITC/propidium iodide (PI) staining kit (Cayman Chemical Company, Ann Arbor, MI) according to the manufacturer’s protocol. Fluorescence intensity was measured by flow cytometry analysis using a BD Accuri^™^ C6 cytometer, with Annexin V-FITC recorded using Ex 488 nm/Em 533/30 nm (FL-1) and PI recorded using Ex 488nm/Em 670LP (FL-3). Measurements were appropriately corrected for spillover using standard color correction procedures.

### Quantitative real-time polymerase chain reaction (qPCR)

qPCR was used to confirm that U87-MG neurospheres exhibit a stem-like phenotype relative to adherent cultures. Cells were grown as neurospheres as described previously for one week, then mRNA was isolated using an Isolate II RNA Mini Kit (Bioline, Taunton, MA). The relative expression of CD133, Nanog, and Sox2 were measured using SensiFAST^™^ SYBR^®^ One-Step master mix and normalized to that of GAPDH. qPCR was performed on a LightCycler^®^ 96 (Roche Diagnostics Corporation, Indianapolis, IN). Primer sequences were as follows: CD133 F - GGACCCATTGGCATTCTC, CD133 R - CAGGACACAGCATAGAATAATC, Nanog F – AAATTGGTGATGAAGATGTATTCG, Nanog R – GCAAAACAGAGCCAAAAACG, Sox2 F – AACATGATGGAGACGGTGCTGAA, Sox2 R – CAGCCGTTCATGTGCGCGTA. Gene expression in cells grown as neurospheres was compared to that of cells grown in adherent conditions.

### Statistical analysis

Differences between two groups were assessed for statistical significance by Student’s *t*-test for each cell line independently. TMZ dose response data and sphere growth data were analyzed using a one-way analysis of variance (ANOVA) with post-hoc Tukey test using MATLAB software (MathWorks, Natick, MA). Neurosphere apoptosis data were analyzed using one-way ANOVA with post-hoc Fisher’s least significant difference test. Flow cytometry data was analyzed using either FlowJo software (Tree Star Inc, Ashland, OR) or CFlow software (Becton Dickinson Biosciences, Franklin Lakes, NJ). Differences were considered statistically significant at *p* < 0.05. Data shown represents the mean ± standard deviation of three independent experimental replicates.

## SUPPLEMENTARY MATERIALS FIGURES



## Abbreviations

GBM: glioblastoma multiforme
TMZ: temozolomide
GSC: glioma stem-like cells
Hh: Hedgehog
Shh: Sonic hedgehog
EdU: 5-ethynyl-2′-deoxyuridine
MGMT: *O*^6^-methylguanine-DNA methyltransferase
