# Supplementary material for: Investigating the role of Hedgehog/GLI1 signaling in glioblastoma cell response to temozolomide
Source: Oncotarget. 2018 Jun 5;9(43):27000–15. doi: 10.18632/oncotarget.25467 (PMC6007474; doi:10.18632/oncotarget.25467)
Supplement: Supplementary file 1 [file oncotarget-09-27000-s001.pdf]

Investigating the role of Hedgehog/GLI1 signaling in glioblastoma cell response to temozolomide

SUPPLEMENTARY MATERIALS

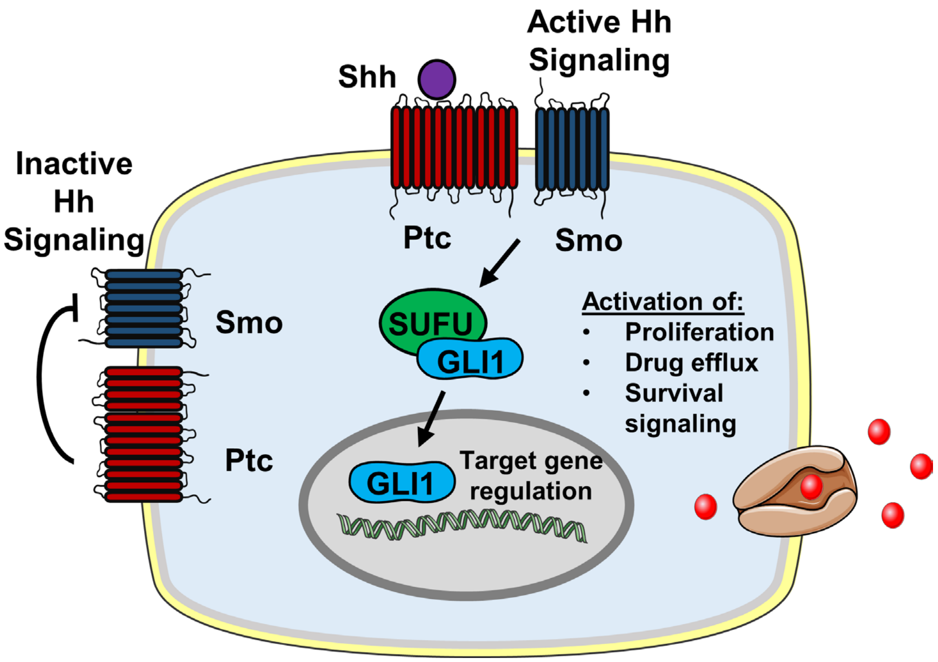

Supplementary Figure 1: Schematic showing a simplified Hedgehog signaling pathway with phenotypic implications in GBM cells.

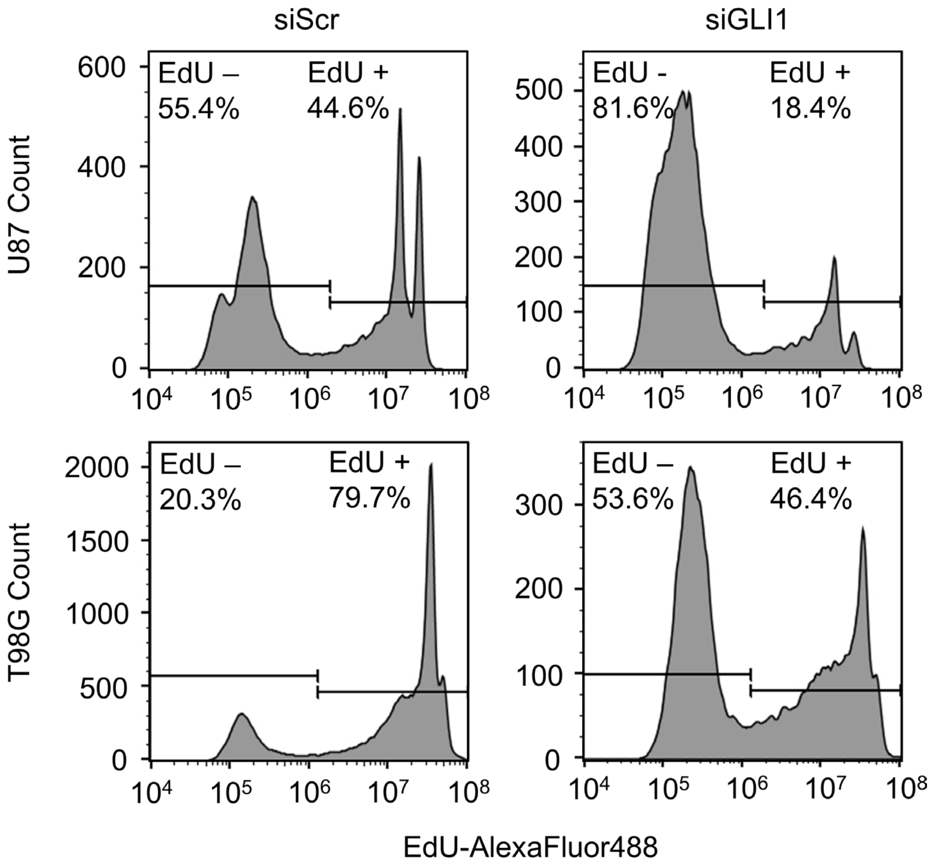

Supplementary Figure 2: Flow cytometric histogram from one representative EdU experiment, summarized in Figure 1D.

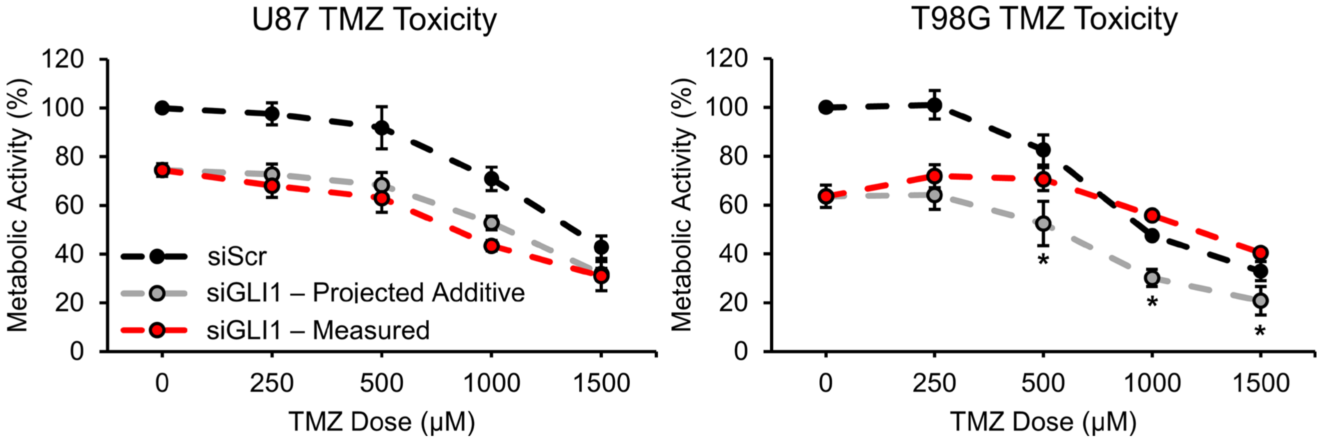

Supplementary Figure 3: Assessment of additive or synergistic effects between GLI1 silencing and TMZ treatment.
$^{*}p < 0.05$ by one-way ANOVA and post-hoc Tukey.

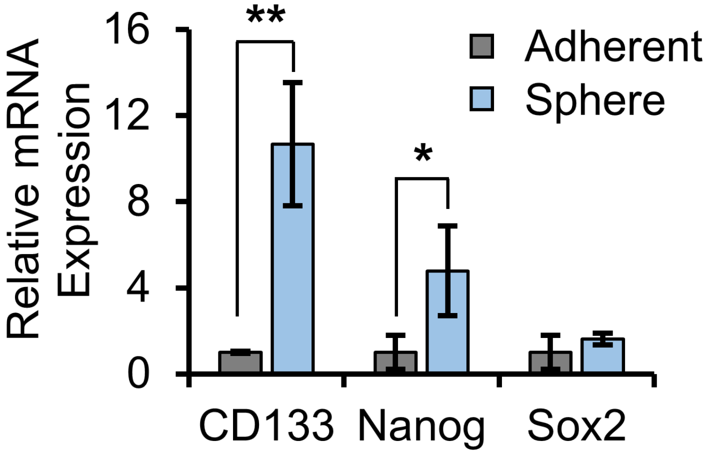

Supplementary Figure 4: By qPCR, growing U87-MG cells in sphere culture increases the expression of genes associated with a stem-like phenotype, $^{**}p < 0.01$, $^{*}p < 0.05$ by Student's *t*-test.

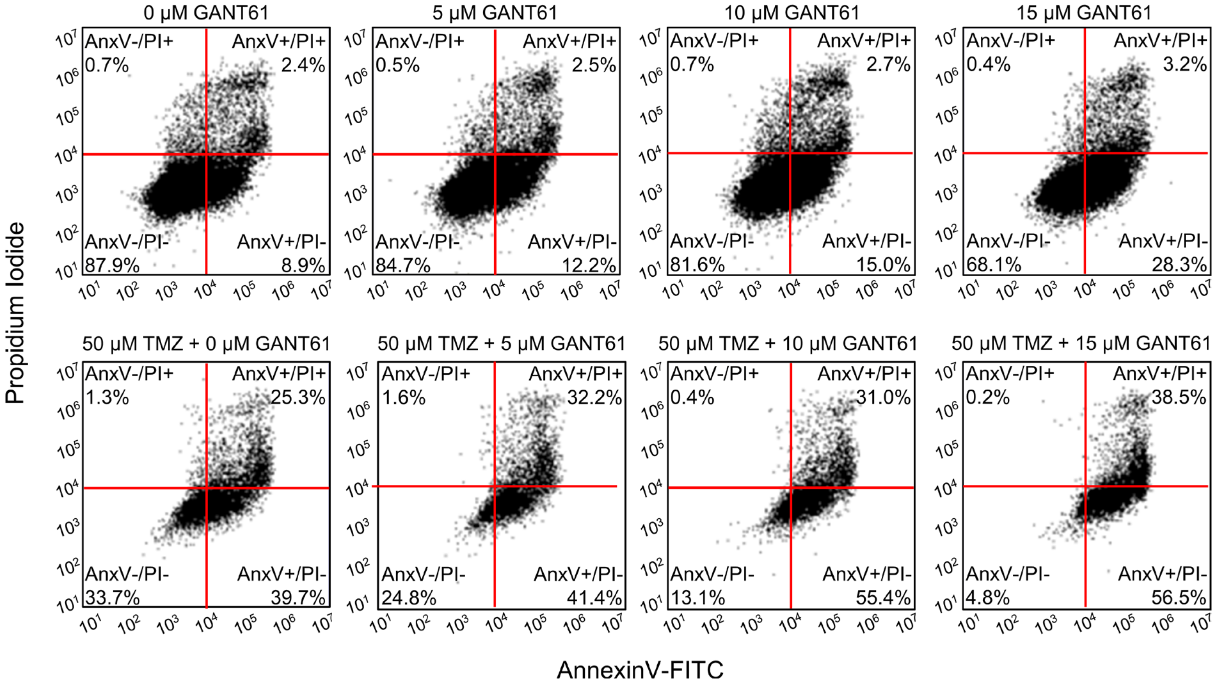

Supplementary Figure 5: Flow scatterplots from one representative run of samples used to assess apoptosis in U87-MG neurospheres, depicting all treatment groups summarized in Figure 4B.

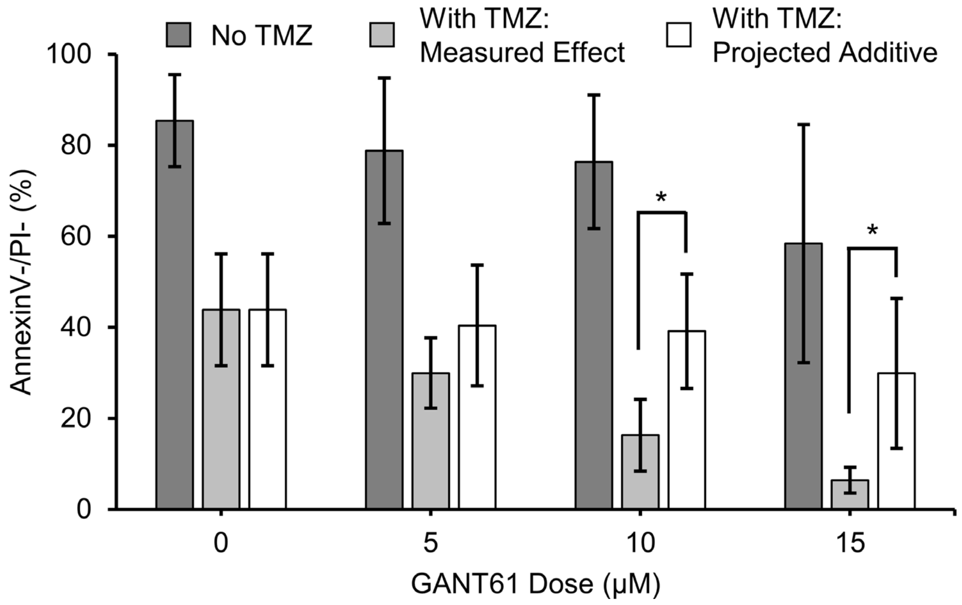

Supplementary Figure 6: Synergy assessment for neurosphere apoptosis in response to co-treatment with TMZ and GANT61. The fraction of measured viable (AnnexinV-, PI-) cells was significantly decreased relative to the projected viability for neurospheres co-treated with TMZ and 10 or 15 µM GANT61. Data shown are means ± standard deviations, *$p < 0.05$ by one-way ANOVA with post-hoc Fisher's least significant difference test.

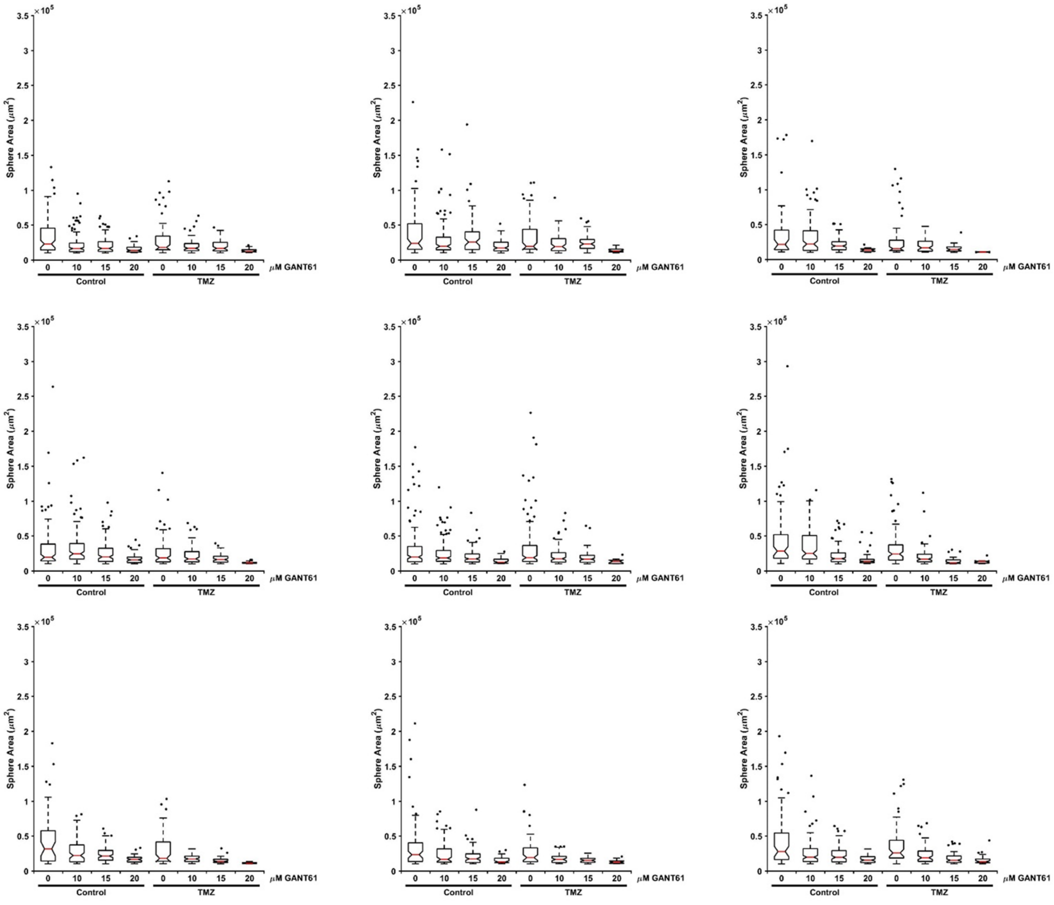

Supplementary Figure 7: Sphere size as determined by automated image analysis in all 9 independent replicates.
